# (*E*)-*N*′-(4-Methoxy­benzyl­idene)thio­phene-2-carbohydrazide

**DOI:** 10.1107/S1600536810017836

**Published:** 2010-05-22

**Authors:** Yu-Feng Li, Fang-Fang Jian

**Affiliations:** a Microscale Science Institute, Department of Chemistry and Chemical Engineering, Weifang University, Weifang 261061, People’s Republic of China; b Microscale Science Institute, Weifang University, Weifang 261061, People’s Republic of China

## Abstract

In the title compound, C_13_H_12_N_2_O_2_S, the dihedral angle between the aromatic rings is 15.20 (11)°. In the crystal, inversion dimers linked by pairs of N—H⋯O hydrogen bonds generate *R*_2_^2^(8) loops.

## Related literature

For a related structure, see: Li & Jian (2010 ▶).
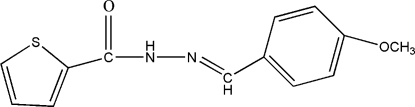


## Experimental

### 

#### Crystal data

C_13_H_12_N_2_O_2_S
*M* *_r_* = 260.31
Monoclinic, 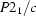

*a* = 16.106 (3) Å
*b* = 5.3292 (11) Å
*c* = 14.812 (3) Å
β = 104.91 (3)°
*V* = 1228.5 (4) Å^3^
*Z* = 4
Mo *K*α radiation
μ = 0.26 mm^−1^
*T* = 293 K
0.25 × 0.22 × 0.18 mm

#### Data collection

Bruker SMART CCD diffractometer
11253 measured reflections
2807 independent reflections
2454 reflections with *I* > 2σ(*I*)
*R*_int_ = 0.027

#### Refinement

*R*[*F* ^2^ > 2σ(*F* ^2^)] = 0.056
*wR*(*F* ^2^) = 0.177
*S* = 1.00
2807 reflections
163 parameters
H-atom parameters constrained
Δρ_max_ = 0.74 e Å^−3^
Δρ_min_ = −0.53 e Å^−3^

### 

Data collection: *SMART* (Bruker 1997 ▶); cell refinement: *SAINT* (Bruker 1997 ▶); data reduction: *SAINT*; program(s) used to solve structure: *SHELXS97* (Sheldrick, 2008 ▶); program(s) used to refine structure: *SHELXL97* (Sheldrick, 2008 ▶); molecular graphics: *SHELXTL* (Sheldrick, 2008 ▶); software used to prepare material for publication: *SHELXTL*.

## Supplementary Material

Crystal structure: contains datablocks global, I. DOI: 10.1107/S1600536810017836/hb5449sup1.cif

Structure factors: contains datablocks I. DOI: 10.1107/S1600536810017836/hb5449Isup2.hkl

Additional supplementary materials:  crystallographic information; 3D view; checkCIF report

## Figures and Tables

**Table 1:** Hydrogen-bond geometry (Å, °)

*D*—H⋯*A*	*D*—H	H⋯*A*	*D*⋯*A*	*D*—H⋯*A*
N1—H1*A*⋯O2^i^	0.86	2.12	2.963 (2)	167

Symmetry code: (i) 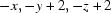.

**Fig. 1.**
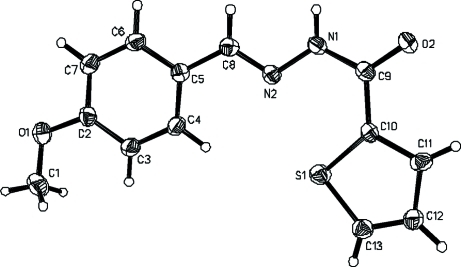
The structure of (I) showing 30% probability displacement ellipsoids.

## supplementary crystallographic information

### Experimental

A mixture of thiophene-2-carbohydrazide (0.10 mol), and 4-methoxybenzaldehyde (0.10 mol) was stirred in refluxing ethanol (10 ml) for 4 h to afford the title compound (0.079 mol, yield 79%). Colourless blocks of (I) were obtained by recrystallization from ethanol at room temperature.

### Refinement

H atoms were fixed geometrically and allowed to ride on their attached atoms, with C—H distances = 0.93-0.97 Å; N—H = 0.86Å and with *U*_iso_(H) = 1.2*U*_eq_(C,*N*) or 1.5*U*_eq_(C_methyl_).

### Crystal data

**Table:**

C_13_H_12_N_2_O_2_S	*F*(000) = 544
*M**_r_* = 260.31	*D*_x_ = 1.407 Mg m^−^^3^
Monoclinic, *P*2_1_/*c*	Mo *K*α radiation, λ = 0.71073 Å
Hall symbol: -P 2ybc	Cell parameters from 2454 reflections
*a* = 16.106 (3) Å	θ = 2.7–25.3°
*b* = 5.3292 (11) Å	µ = 0.26 mm^−^^1^
*c* = 14.812 (3) Å	*T* = 293 K
β = 104.91 (3)°	Block, colorless
*V* = 1228.5 (4) Å^3^	0.25 × 0.22 × 0.18 mm
*Z* = 4	

### Data collection

**Table:**

Bruker SMART CCD diffractometer	2454 reflections with *I* > 2σ(*I*)
Radiation source: fine-focus sealed tube	*R*_int_ = 0.027
graphite	θ_max_ = 27.5°, θ_min_ = 3.3°
phi and ω scans	*h* = −20→20
11253 measured reflections	*k* = −6→6
2807 independent reflections	*l* = −19→18

### Refinement

**Table:**

Refinement on *F*^2^	Primary atom site location: structure-invariant direct methods
Least-squares matrix: full	Secondary atom site location: difference Fourier map
*R*[*F*^2^ > 2σ(*F*^2^)] = 0.056	Hydrogen site location: inferred from neighbouring sites
*wR*(*F*^2^) = 0.177	H-atom parameters constrained
*S* = 1.00	*w* = 1/[σ^2^(*F*_o_^2^) + (0.1142*P*)^2^ + 0.7797*P*] where *P* = (*F*_o_^2^ + 2*F*_c_^2^)/3
2807 reflections	(Δ/σ)_max_ = 0.001
163 parameters	Δρ_max_ = 0.74 e Å^−^^3^
0 restraints	Δρ_min_ = −0.53 e Å^−^^3^

### Special details

**Table:**

Geometry. All esds (except the esd in the dihedral angle between two l.s. planes) are estimated using the full covariance matrix. The cell esds are taken into account individually in the estimation of esds in distances, angles and torsion angles; correlations between esds in cell parameters are only used when they are defined by crystal symmetry. An approximate (isotropic) treatment of cell esds is used for estimating esds involving l.s. planes.
Refinement. Refinement of *F*^2^ against ALL reflections. The weighted *R*-factor wR and goodness of fit *S* are based on *F*^2^, conventional *R*-factors *R* are based on *F*, with *F* set to zero for negative *F*^2^. The threshold expression of *F*^2^ > σ(*F*^2^) is used only for calculating *R*-factors(gt) etc. and is not relevant to the choice of reflections for refinement. *R*-factors based on *F*^2^ are statistically about twice as large as those based on *F*, and *R*- factors based on ALL data will be even larger.

### Fractional atomic coordinates and isotropic or equivalent isotropic displacement parameters (Å^2^)

**Table:**

	*x*	*y*	*z*	*U*_iso_*/*U*_eq_	
S1	0.19516 (4)	0.31168 (11)	0.98996 (4)	0.0456 (2)	
N2	0.17079 (11)	0.7123 (3)	1.09840 (12)	0.0364 (4)	
N1	0.09320 (10)	0.7857 (3)	1.04270 (12)	0.0372 (4)	
H1A	0.0643	0.9002	1.0620	0.045*	
O2	−0.00677 (9)	0.7645 (3)	0.90675 (11)	0.0437 (4)	
C9	0.06111 (12)	0.6815 (4)	0.95773 (14)	0.0347 (4)	
C8	0.19896 (13)	0.8393 (4)	1.17289 (15)	0.0381 (4)	
H8A	0.1659	0.9700	1.1865	0.046*	
C5	0.28237 (13)	0.7838 (4)	1.23759 (14)	0.0366 (4)	
O1	0.52295 (11)	0.6947 (4)	1.42184 (13)	0.0608 (5)	
C11	0.07350 (14)	0.3690 (4)	0.83349 (18)	0.0449 (5)	
H11A	0.0269	0.4297	0.7878	0.054*	
C10	0.10555 (11)	0.4692 (4)	0.92598 (13)	0.0344 (4)	
C2	0.44404 (14)	0.7112 (4)	1.35954 (15)	0.0428 (5)	
C3	0.41450 (15)	0.5483 (5)	1.28526 (16)	0.0480 (5)	
H3A	0.4485	0.4149	1.2758	0.058*	
C6	0.31271 (15)	0.9418 (5)	1.31370 (16)	0.0473 (5)	
H6A	0.2785	1.0739	1.3239	0.057*	
C7	0.39260 (16)	0.9065 (5)	1.37430 (17)	0.0520 (6)	
H7A	0.4118	1.0136	1.4249	0.062*	
C4	0.33415 (15)	0.5854 (4)	1.22531 (16)	0.0454 (5)	
H4A	0.3144	0.4752	1.1757	0.054*	
C13	0.19081 (14)	0.1074 (4)	0.90128 (16)	0.0447 (5)	
H13A	0.2289	−0.0259	0.9053	0.054*	
C12	0.12683 (14)	0.1562 (5)	0.82410 (17)	0.0457 (5)	
H12A	0.1178	0.0615	0.7697	0.055*	
C1	0.57849 (17)	0.5001 (7)	1.4092 (2)	0.0675 (8)	
H1B	0.6315	0.5105	1.4569	0.101*	
H1C	0.5898	0.5168	1.3489	0.101*	
H1D	0.5518	0.3407	1.4131	0.101*	

### Atomic displacement parameters (Å^2^)

**Table:**

	*U*^11^	*U*^22^	*U*^33^	*U*^12^	*U*^13^	*U*^23^
S1	0.0484 (4)	0.0437 (4)	0.0437 (3)	0.0088 (2)	0.0098 (2)	0.0038 (2)
N2	0.0340 (8)	0.0363 (9)	0.0375 (9)	0.0022 (6)	0.0069 (7)	0.0034 (7)
N1	0.0319 (8)	0.0382 (9)	0.0407 (9)	0.0047 (6)	0.0079 (7)	0.0012 (7)
O2	0.0310 (7)	0.0480 (9)	0.0477 (9)	0.0055 (6)	0.0019 (6)	0.0001 (7)
C9	0.0301 (9)	0.0343 (10)	0.0404 (10)	−0.0017 (7)	0.0103 (7)	0.0048 (7)
C8	0.0390 (10)	0.0389 (10)	0.0375 (10)	0.0040 (8)	0.0122 (8)	0.0011 (8)
C5	0.0389 (10)	0.0369 (10)	0.0343 (10)	0.0005 (8)	0.0099 (8)	0.0016 (7)
O1	0.0453 (9)	0.0744 (13)	0.0518 (10)	0.0062 (8)	−0.0072 (7)	−0.0129 (9)
C11	0.0408 (11)	0.0411 (11)	0.0596 (13)	−0.0091 (9)	0.0253 (10)	−0.0152 (10)
C10	0.0287 (8)	0.0361 (10)	0.0380 (9)	−0.0016 (7)	0.0079 (7)	0.0030 (8)
C2	0.0385 (10)	0.0487 (12)	0.0376 (10)	−0.0005 (9)	0.0033 (8)	−0.0016 (9)
C3	0.0462 (12)	0.0447 (12)	0.0479 (12)	0.0092 (9)	0.0030 (9)	−0.0065 (10)
C6	0.0508 (12)	0.0478 (12)	0.0417 (11)	0.0087 (10)	0.0091 (9)	−0.0097 (9)
C7	0.0542 (13)	0.0543 (14)	0.0423 (11)	0.0024 (11)	0.0029 (10)	−0.0153 (10)
C4	0.0477 (11)	0.0401 (11)	0.0422 (11)	0.0043 (9)	0.0005 (9)	−0.0084 (9)
C13	0.0442 (11)	0.0382 (11)	0.0525 (12)	0.0007 (9)	0.0142 (9)	−0.0021 (9)
C12	0.0386 (11)	0.0480 (12)	0.0505 (12)	−0.0069 (9)	0.0116 (9)	−0.0118 (10)
C1	0.0423 (13)	0.086 (2)	0.0669 (17)	0.0125 (13)	0.0002 (11)	−0.0018 (15)

### Geometric parameters (Å, °)

**Table:**

S1—C13	1.694 (2)	C11—H11A	0.9300
S1—C10	1.727 (2)	C2—C7	1.382 (3)
N2—C8	1.274 (3)	C2—C3	1.386 (3)
N2—N1	1.366 (2)	C3—C4	1.382 (3)
N1—C9	1.351 (3)	C3—H3A	0.9300
N1—H1A	0.8600	C6—C7	1.379 (3)
O2—C9	1.239 (2)	C6—H6A	0.9300
C9—C10	1.479 (3)	C7—H7A	0.9300
C8—C5	1.466 (3)	C4—H4A	0.9300
C8—H8A	0.9300	C13—C12	1.353 (3)
C5—C4	1.387 (3)	C13—H13A	0.9300
C5—C6	1.391 (3)	C12—H12A	0.9300
O1—C2	1.368 (3)	C1—H1B	0.9600
O1—C1	1.413 (3)	C1—H1C	0.9600
C11—C10	1.437 (3)	C1—H1D	0.9600
C11—C12	1.451 (3)		
			
C13—S1—C10	91.43 (11)	C4—C3—C2	119.6 (2)
C8—N2—N1	115.96 (18)	C4—C3—H3A	120.2
C9—N1—N2	121.10 (17)	C2—C3—H3A	120.2
C9—N1—H1A	119.4	C7—C6—C5	121.3 (2)
N2—N1—H1A	119.4	C7—C6—H6A	119.4
O2—C9—N1	119.15 (19)	C5—C6—H6A	119.4
O2—C9—C10	120.17 (19)	C6—C7—C2	119.8 (2)
N1—C9—C10	120.68 (17)	C6—C7—H7A	120.1
N2—C8—C5	121.42 (19)	C2—C7—H7A	120.1
N2—C8—H8A	119.3	C3—C4—C5	121.3 (2)
C5—C8—H8A	119.3	C3—C4—H4A	119.3
C4—C5—C6	118.0 (2)	C5—C4—H4A	119.3
C4—C5—C8	123.29 (19)	C12—C13—S1	113.72 (18)
C6—C5—C8	118.65 (19)	C12—C13—H13A	123.1
C2—O1—C1	117.9 (2)	S1—C13—H13A	123.1
C10—C11—C12	107.8 (2)	C13—C12—C11	114.2 (2)
C10—C11—H11A	126.1	C13—C12—H12A	122.9
C12—C11—H11A	126.1	C11—C12—H12A	122.9
C11—C10—C9	120.20 (18)	O1—C1—H1B	109.5
C11—C10—S1	112.76 (16)	O1—C1—H1C	109.5
C9—C10—S1	127.02 (15)	H1B—C1—H1C	109.5
O1—C2—C7	115.6 (2)	O1—C1—H1D	109.5
O1—C2—C3	124.4 (2)	H1B—C1—H1D	109.5
C7—C2—C3	120.0 (2)	H1C—C1—H1D	109.5

### Hydrogen-bond geometry (Å, °)

**Table:**

*D*—H···*A*	*D*—H	H···*A*	*D*···*A*	*D*—H···*A*
N1—H1A···O2^i^	0.86	2.12	2.963 (2)	167

Symmetry codes: (i) −*x*, −*y*+2, −*z*+2.

## References

[bb1] Bruker (1997). *SMART* and *SAINT* Bruker AXS, Inc., Madison, Wisconsin, USA.

[bb2] Li, Y.-F. & Jian, F.-F. (2010). Acta Cryst. E**66**, o1398. 10.1107/S1600536810017976 PMC2979519 21579477

[bb3] Sheldrick, G. M. (2008). *Acta Cryst.* A**64**, 112–122. 10.1107/S0108767307043930 18156677

